# Future Projections of Biodiversity Under Global Change Need to Include Genetic Diversity

**DOI:** 10.1111/gcb.70477

**Published:** 2025-08-26

**Authors:** Roslyn C. Henry

**Affiliations:** ^1^ School of Biological Sciences University of Aberdeen, King's College Aberdeen UK

**Keywords:** biodiversity, forecasting, genetic diversity, macrogenetics, modeling, projections

## Abstract

This perspective argues that current methods for predicting biodiversity loss from future land use and climate change models are incomplete without incorporating projections of genetic diversity. Without methods to estimate current and future changes in genetic diversity, we cannot fully anticipate extinction risk, nor can we measure progress toward conservation targets. This oversight threatens to undermine our most ambitious biodiversity goals. We need a vanguard shift in how forecasting is approached, one that integrates genetic data into global biodiversity models.

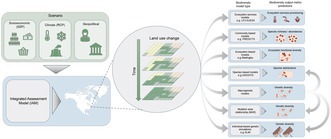

A critical blind spot persists in biodiversity forecasting. Although international policy has appropriately prioritized halting and reversing biodiversity loss, the tools used to predict that loss remain incomplete without including estimates of present and future global genetic diversity. Even the most recent and comprehensive scenario‐based approaches, such as those by Pereira et al. (2024), which integrate Shared Socioeconomic Pathways (SSPs) with Representative Concentration Pathways (RCPs) to model changes in biodiversity and ecosystem services across multiple frameworks, do not project changes in genetic diversity. Without methods to estimate current and project future changes in genetic diversity, we cannot fully anticipate extinction risk, nor can we measure progress toward conservation targets. This oversight threatens to undermine our most ambitious biodiversity goals.

Genetic diversity matters because it determines a species' capacity to adapt, persist, and recover. Climate and land use change can rapidly deplete genetic variation, sometimes more drastically than they reduce population size. While not always immediately visible, the depletion of genetic diversity sets the stage for extinction debts, that is, delayed biodiversity losses that will manifest in the future.

The neglect of genetic diversity in forecasting is not accidental: it reflects scarce genetic data (Leigh et al. 2021) and expensive technologies, leading to underdeveloped methods and a historical lack of integration between geneticists and conservation practitioners (Hoban et al. 2023). Until recently, policy frameworks offered little incentive for integrating genetic indicators in projections, especially for wild species. The previous CBD Aichi targets, for instance, focused largely on domesticated species. As a result, genetic monitoring for wild species has garnered limited investment. Without such data collection, our ability to develop methods for estimating current and projecting future trajectories of genetic diversity has been restricted.

That is now changing. The Kunming‐Montreal Global Biodiversity Framework (GBF) explicitly includes genetic diversity in its 2050 targets, signaling a shift in priorities. Meanwhile, advances in genomic methods, data availability, and modeling tools provide new opportunities to address this long‐standing omission. Such improvements suggest that now is the time to develop a holistic biodiversity forecasting framework that incorporates both species and genetic diversity under global change (Exposito‐Alonso et al. 2022). Where climate forecasting, through the IPCC, has long guided international action, biodiversity forecasting remains comparatively underdeveloped. The Intergovernmental Science‐Policy Platform on Biodiversity and Ecosystem Services (IPBES) has noted low confidence in biodiversity projections. Including genetic diversity will improve such projections but also enhance progress toward GBF targets. Moreover, recent research shows that IUCN Red List status, based on demographic data, poorly reflects genetic status (Schmidt et al. 2023). Including genetic data in forecasting efforts would provide a more complete picture of species' resilience and risk, enhancing our ability to anticipate and mitigate biodiversity loss.

To meet the moment, biodiversity forecasting must evolve. We need a vanguard shift in how forecasting is approached, one that integrates genetic data into global biodiversity models. This is not a minor adjustment, but a necessary reimagining of the science and methods needed to guide conservation in the 21st century. Incorporating macrogenetics, the mutations‐area relationship (MAR), and individual‐based genetic simulations would establish a comprehensive framework for biodiversity forecasting under global change.

Moving beyond species‐level estimates of biodiversity change to include genetic diversity could be achieved through the emerging field of macrogenetics. Macrogenetics examines genetic diversity at broad scales, often across large spatial, temporal, or taxonomic extents (Leigh et al. 2021). While species‐level biodiversity forecasts often rely on statistical relationships between drivers like climate or land‐use change and species abundance or richness (Pereira et al. 2024), macrogenetics seeks to establish similar relationships between anthropogenic drivers and genetic diversity. This enables predictions of environmental change impacts, even for species or populations with limited genetic data. The strength of macrogenetics lies in its ability to leverage existing data to estimate genetic responses for under‐studied species or populations (Leigh et al. 2021).

Macrogenetic approaches have used genetic marker data across species to estimate current genetic diversity loss attributed to human population growth and anthropogenic land use change (Millette et al. 2020) but have not yet been included in future forecasts. By describing relationships between environmental drivers and genetic indicators, macrogenetics offers the potential to create more comprehensive biodiversity change projections, from the gene to species level (Figure 1). For example, high‐resolution maps highlighting regions crucial for genetic diversity could complement species‐level conservation planning in the context of anthropogenic pressures (Exposito‐Alonso et al. 2022).

**FIGURE 1:**
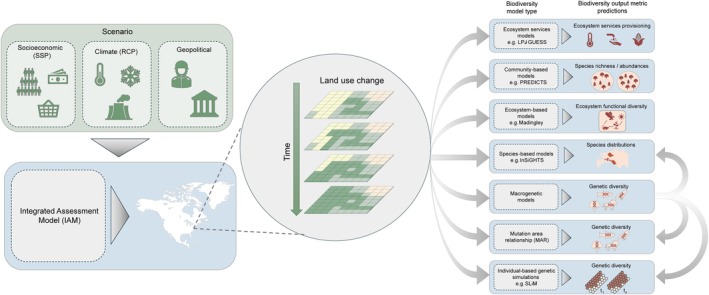
A proposed holistic framework to predict biodiversity change across multiple facets. This includes species‐level biodiversity responses, ecosystem services, and genetic diversity responses. Global change scenarios with different climate, socioeconomic, and/or geopolitical pathways are developed and incorporated into land use models. Land use change through time is projected, and data from such are used within biodiversity modeling approaches to predict changes in different facets of biodiversity, including genetic diversity. The outputs from these approaches are complementary, providing insights that can refine and inform one another. Map lines delineate example areas and do not necessarily depict accepted national boundaries.

Findings from macrogenetics studies on the impacts of human activity on current genetic diversity are mixed. One study across 91 species estimated that approximately 6% of genetic diversity has been lost since the Industrial Revolution (Leigh et al. 2019), while a more recent study found no significant impact of human activity on global genetic diversity between 1980 and 2016 (Millette et al. 2020). While the reasons for these discrepancies remain unclear, they underscore the urgent need for further research to reconcile the contrasting results of early genetic forecasting studies, allowing for more reliable projections of future genetic diversity. Macrogenetics is still a relatively new field and faces challenges, such as sensitivity to genetic markers and the potential underestimation of genetic loss due to pre‐existing ecosystem degradation, yet it is poised to grow stronger as genomic technologies rapidly advance, increasing both the volume and variety of genetic data available (Leigh et al. 2021).

A further emerging approach to forecasting genetic diversity uses theoretical models as a complement to traditional macrogenetics. The mutation–area relationship (MAR, Exposito‐Alonso et al. 2022), analogous to the species–area relationship (SAR), predicts genetic diversity loss with habitat reduction via a power law, offering a tractable framework for estimating genetic erosion. MAR shows promise for anticipating intraspecific genetic threats under global change but remains largely untested. Its predictive accuracy depends on species‐specific traits such as dispersal and mating behavior, highlighting the need for broader application across taxa and ecosystems.

A more detailed, process‐based option is individual‐based, forward‐time modeling (IBMs), which simulates how demographic and evolutionary processes shape genetic diversity within and between populations over time. Well‐suited to non‐equilibrium systems, IBMs can explore genetic consequences of dynamic environmental change but are typically limited to single species or populations, hindering generalization. High data and computational demands necessitate simplifying assumptions, which may reduce realism. Balancing model complexity with interpretability remains a challenge, but when parameterized with empirical data and applied efficiently, IBMs can yield valuable insights into the temporal dynamics of genetic diversity under anthropogenic change.

Rather than viewing macrogenetic forecasting, MAR, and individual‐based simulations as competing approaches, they should be seen as complementary. MAR offers broad, scalable estimates useful for global assessments, while individual‐based models provide depth and mechanistic insight at finer scales. Together, they represent critical tools in the development of robust, multi‐scale forecasts of genetic change. Moreover, both MAR and IBMs can incorporate macrogenetic data to inform predictions, as demonstrated in previous studies (Exposito‐Alonso et al. 2022). The framework in Figure 1 envisions macrogenetics as a tool to directly link broad‐scale emergent genetic patterns to biodiversity outcomes, while also serving as an indirect input for parameterizing models such as MAR or fine‐scale process‐based individual‐based models. Additionally, macrogenetic data can enhance species distribution models (SDMs) by incorporating genetic diversity as a predictor, such as by inferring gene flow, to improve estimates of species dispersal potential and refine range predictions.

Enhancing our ability to forecast genetic diversity in response to climate and land‐use changes presents significant but surmountable challenges. A key hurdle has been identifying reliable genetic indicators to link genetic diversity with anthropogenic drivers in future scenarios (Hoban et al. 2022, 2023; Laikre et al. 2020). To address this, the Group on Earth Observations Biodiversity Observation Network (GEO BON) introduced genetic Essential Biodiversity Variables (EBVs); standardized and scalable metrics that track biodiversity changes across space and time. If limitations, such as a lack of sensitivity to detecting changes and data biases, can be addressed, then genetic EBVs could provide a more comprehensive and accessible measure of genetic diversity (Hoban et al. 2022). Additionally, advancements in genome sequencing and adherence to (FAIR) data principles continue to expand the availability of relevant datasets and could improve EBV calculations (Wilkinson et al. 2016). These improvements, combined with the development of methodologies to better understand the relationship between anthropogenic impacts and genetic diversity, are paving the way for increasingly robust macrogenetic and theoretical approaches (Leigh et al. 2021).

By leveraging cutting‐edge macrogenetic and theoretical models, now is the time to explore how human‐induced changes, such as climate change, or conservation strategies might influence genetic diversity. This expanded scope will allow for more accurate forecasts that go beyond purely demographic projections, capturing both immediate and long‐term risks to biodiversity. These forecasts will be critical not only for accelerating progress toward specific conservation targets, but also for informing broader international commitments. They will equip researchers, conservation practitioners, and policymakers with critical insights to advance Sustainable Development Goals (SDGs) such as SDG 15 (Life on Land), SDG 13 (Climate Action), and SDG 2 (Zero Hunger), all of which depend on healthy, resilient ecosystems. Genetic diversity not only underpins ecological stability but also delivers socio‐economic benefits, such as improved crop yields. As such, by anticipating areas of genetic vulnerability and resilience, this approach can guide conservation strategies, strengthen food and livelihood security, and ensure the long‐term sustainability of both natural and human systems. In this way, a genomically informed forecasting framework offers a powerful tool to inform biodiversity science and global policy agendas for the benefit of both people and nature.

## Author Contributions

**Roslyn C. Henry:** conceptualization, writing – original draft, writing – review and editing.

## Conflicts of Interest

The author declares no conflicts of interest.

## Data Availability

The author has nothing to report.
